# Assessment of a Deep Learning Model to Predict Hepatocellular Carcinoma in Patients With Hepatitis C Cirrhosis

**DOI:** 10.1001/jamanetworkopen.2020.15626

**Published:** 2020-09-01

**Authors:** George N. Ioannou, Weijing Tang, Lauren A. Beste, Monica A. Tincopa, Grace L. Su, Tony Van, Elliot B. Tapper, Amit G. Singal, Ji Zhu, Akbar K. Waljee

**Affiliations:** 1 Division of Gastroenterology, Department of Medicine, Veterans Affairs Puget Sound Healthcare System and University of Washington, Seattle; 2 Research and Development, Veterans Affairs Puget Sound Healthcare System, Seattle, Washington; 3 Department of Statistics, University of Michigan, Ann Arbor; 4 Division of General Internal Medicine, Department of Medicine, Veterans Affairs Puget Sound Healthcare System and University of Washington, Seattle; 5 Michigan Medicine, Division of Gastroenterology and Hepatology, Department of Internal Medicine, Ann Arbor; 6 VA Ann Arbor Health Services Research and Development Center of Clinical Management Research, Ann Arbor, Michigan; 7 Michigan Integrated Center for Health Analytics and Medical Prediction (MiCHAMP), Ann Arbor; 8 Division of Gastroenterology, Department of Medicine, University of Texas Southwestern, Dallas

## Abstract

**Importance:**

Deep learning, a family of machine learning models that use artificial neural networks, has achieved great success at predicting outcomes in nonmedical domains.

**Objective:**

To examine whether deep learning recurrent neural network (RNN) models that use raw longitudinal data extracted directly from electronic health records outperform conventional regression models in predicting the risk of developing hepatocellular carcinoma (HCC).

**Design, Setting, and Participants:**

This prognostic study included 48 151 patients with hepatitis C virus (HCV)–related cirrhosis in the national Veterans Health Administration who had at least 3 years of follow-up after the diagnosis of cirrhosis. Patients were identified by having at least 1 positive HCV RNA test between January 1, 2000, to January 1, 2016, and were followed up from the diagnosis of cirrhosis to January 1, 2019, for the development of incident HCC. A total of 3 models predicting HCC during a 3-year period were developed and compared, as follows: (1) logistic regression (LR) with cross-sectional inputs (cross-sectional LR); (2) LR with longitudinal inputs (longitudinal LR); and (3) RNN with longitudinal inputs. Data analysis was conducted from April 2018 to August 2020.

**Exposures:**

Development of HCC.

**Main Outcomes and Measures:**

Area under the receiver operating characteristic curve, area under the precision-recall curve, and Brier score.

**Results:**

During a mean (SD) follow-up of 11.6 (5.0) years, 10 741 of 48 151 patients (22.3%) developed HCC (annual incidence, 3.1%), and a total of 52 983 samples (51 948 [98.0%] from men) were collected. Patients who developed HCC within 3 years were older than patients who did not (mean [SD] age, 58.2 [6.6] years vs 56.9 [6.9] years). RNN models had superior mean (SD) area under the receiver operating characteristic curve (0.759 [0.009]) and mean (SD) Brier score (0.136 [0.003]) than cross-sectional LR (0.689 [0.009] and 0.149 [0.003], respectively) and longitudinal LR (0.682 [0.007] and 0.150 [0.003], respectively) models. Using the RNN model, the samples with the mean (SD) highest 51% (1.5%) of HCC risk, in which 80% of all HCCs occurred, or the mean (SD) highest 66% (1.2%) of HCC risk, in which 90% of all HCCs occurred, could potentially be targeted. Among samples from patients who achieved sustained virologic response, the performance of the RNN models was even better (mean [SD] area under receiver operating characteristic curve, 0.806 [0.025]; mean [SD] Brier score, 0.117 [0.007]).

**Conclusions and Relevance:**

In this study, deep learning RNN models outperformed conventional LR models, suggesting that RNN models could be used to identify patients with HCV-related cirrhosis with a high risk of developing HCC for risk-based HCC outreach and surveillance strategies.

## Key Points

**Question:**

Can deep learning recurrent neural network (RNN) models using raw longitudinal data extracted directly from electronic health records outperform conventional regression models in predicting the risk of developing hepatocellular carcinoma (HCC)?

**Findings:**

This prognostic study included 48 151 patients with hepatitis C virus (HCV)–related cirrhosis in the national Veterans Health Administration who had at least 3 years of follow-up after the diagnosis of cirrhosis. Deep learning RNN models outperformed conventional linear regression models and could be used to identify patients with HCV-related cirrhosis at high risk of developing HCC.

**Meaning:**

The findings of this study suggest that RNN models could have multiple applications in clinical practice and could be applied to HCC outreach and surveillance strategies.

## Teaser

This prognostic study examines whether deep learning recurrent neural network (RNN) models that use raw longitudinal data extracted directly from electronic health records outperform conventional regression models in predicting the risk of developing hepatocellular carcinoma (HCC).

## Introduction

Patients with chronic hepatitis C virus (HCV) infection have a high risk of developing hepatocellular carcinoma (HCC). The risk of HCC increases among patients with HCV infection when they develop advanced fibrosis or cirrhosis. Conversely, the risk decreases after HCV eradication,^1,2,3,4,5^ which is becoming increasingly common. Many other factors are known to be associated with increased risk of HCC among patients with HCV or cirrhosis, including low platelet count, increased aspartate transaminase–to–alanine aminotransferase (AST:ALT) ratio, male sex, and older age.^6^ Among patients with HCV-related cirrhosis, the annual risk of HCC varies from less than 1% to more than 5%, depending on a number of readily available, well-described risk factors. Conventional regression models have recently been developed to estimate the risk of HCC in patients with HCV according to the presence or absence of cirrhosis, response to antiviral treatment, and a small number of routinely available baseline clinical characteristics.^6^

HCC risk prediction in patients with HCV infection is particularly difficult because it can fluctuate over time. The development of cirrhosis and the eradication of HCV represent major transition points at which HCC risk changes drastically and abruptly. In addition, HCC risk changes more gradually as patients age or as portal hypertension worsens, liver stiffness increases, or platelet count declines over a period of years. Therefore, longitudinal models that can adequately capture the changes in these predictive factors over time could be ideally suited for HCC risk prediction. Recent advances in deep learning models have been shown to help learn feature representations of data and improve model performance in different domains, such as computer vision and natural language processing. Deep learning models have also been successfully applied to health care to predict clinical events,^7^ disease classification,^8^ and electronic health record (EHR) data augmentation.^9^ Among them, various types of model architectures have been used, such as feedforward neural networks, recurrent neural networks (RNNs), and convolutional neural networks.^10^ The recurrent mechanism used in RNNs can help capture temporal dynamics and long-term information over time; RNNs can also handle longitudinal data with varying lengths of follow-up. Therefore, RNNs are powerful and popular models for processing sequential data, such as time series, longitudinal clinical events, and clinician’s text notes. Our aim was to develop and examine the prediction accuracy of a deep learning model based on RNNs for predicting progression to HCC in a cohort of patients with chronic HCV infection in the Veterans Health Administration (VHA). Furthermore, we aimed to compare the performance of a deep learning RNN model with conventional logistic regression (LR) models.

## Methods

### Data Source

The VHA is the largest integrated health care system treating patients with HCV in the United States.^11^ The VHA uses a single comprehensive electronic health care information network that integrates all care applications into a single, common database. We obtained data on all patients with chronic HCV in the VHA system using the Corporate Data Warehouse, a national, continually updated repository of health care data.^12^ The study was approved by the institutional review boards of the Puget Sound and Ann Arbor VA Healthcare Systems. Patient consent was waived based on the utility of secondary data analysis given the number of patients involved. This study followed the Transparent Reporting of a Multivariable Prediction Model for Individual Prognosis or Diagnosis (TRIPOD) reporting guideline.

### Study Population

Using VHA Corporate Data Warehouse data, we identified 280 418 patients with at least 1 positive HCV RNA test during the 16-year period from January 1, 2000, to January 1, 2016, and retrospectively followed up their EHRs in the VHA system until January 1, 2019. We excluded 203 573 patients (72.6%) who were never diagnosed with cirrhosis because HCC screening is only recommended for patients with HCV after they develop advanced fibrosis or cirrhosis; we excluded an additional 3680 patients (1.3%) in whom the diagnosis of HCC preceded the diagnosis of cirrhosis. The diagnosis of cirrhosis was based on the presence of the *International Classification of Diseases, Ninth Revision *(*ICD*-*9*) or *International Statistical Classification of Diseases and Related Health Problems, Tenth Revision *(*ICD-10*) codes for cirrhosis or complications of cirrhosis (ie, gastroesophageal varices, encephalopathy, nonmalignant ascites, hepatorenal syndrome, hepatopulmonary syndrome) (eTable 1 in the Supplement), recorded at least twice in any inpatient or outpatient encounter. This approach has been validated and widely used in VHA-based studies by us^4,13,14,15,16,17,18,19,20^ and others.^21,22,23^ The earliest date that any of these *ICD*-*9* or *ICD*-*10* codes were recorded was considered the date of cirrhosis diagnosis. Finally, because our aim was to develop longitudinal models predicting the development of HCC during a 3-year period, we excluded 25 014 patients (8.9%) who had less than 3 years of available follow-up from the diagnosis of cirrhosis to their last visit in the VA system. This resulted in a final analytic sample of 48 151 patients with HCV-related cirrhosis and at least 3 years of follow-up after the diagnosis of cirrhosis, of whom 10 741 (22.3%) developed HCC during follow-up.

### Diagnosis of HCC

The diagnosis of HCC was based on the presence of *ICD*-*9* code 155.0 or *ICD*-*10* code C22.0 (the VHA began using *ICD*-*10* codes on October 1, 2015), recorded at least twice. The *ICD*-*9* code–based definition of HCC using VHA records has been shown to have a positive predictive value of 84% to 94% compared with medical record extraction^23,24,25^ and has been widely used by us^17,18,20,26^ and other investigators.^27,28,29^

### Predictor Variables

We used 2 types of predictor variables for HCC prediction, as follows: (1) 4 baseline predictors, which do not change over time, ie, age at cirrhosis diagnosis, sex, race, and HCV genotype; and (2) 27 longitudinal predictors, which may change over time and are available at multiple times during follow-up, including development of cirrhosis, achievement of sustained virologic response (SVR), body mass index (calculated as weight in kilograms divided by height in meters squared), and 24 laboratory blood tests (bilirubin, AST, AST–upper limit of normal (ULN) ratio, ALT, ALT:ULN ratio, α-fetoprotein, α-fetoprotein–ULN ratio, alkaline phosphatase, alkaline phosphatase–ULN ratio, albumin, AST:ALT ratio, fibrosis-4 (FIB-4) score,^30^ AST-platelet ratio index (APRI), blood urea nitrogen, creatinine, glucose, international normalized ratio, hemoglobin, white blood cell count, platelet count, sodium, potassium, chloride, and total protein). The development of cirrhosis is a longitudinal indicator, which starts with 0 and changes to 1 at the date of diagnosis of cirrhosis. Therefore, it indicates not only whether a patient developed cirrhosis but also how long the patient had cirrhosis. To determine SVR, we identified all antiviral treatment regimens (interferon and/or direct-acting antiviral agents) and whether they resulted in SVR, defined as a serum HCV RNA viral load below the lower limit of detection performed at least 12 weeks after the end of treatment.^31^ Also, to identify any patients who might have achieved SVR as a result of antiviral treatment received outside the VHA, we defined SVR in patients with a prior positive HCV viral load who had subsequent persistent negative viral loads.

### Rationale for Cases and Controls Used in Model Building

**Figure 1.**
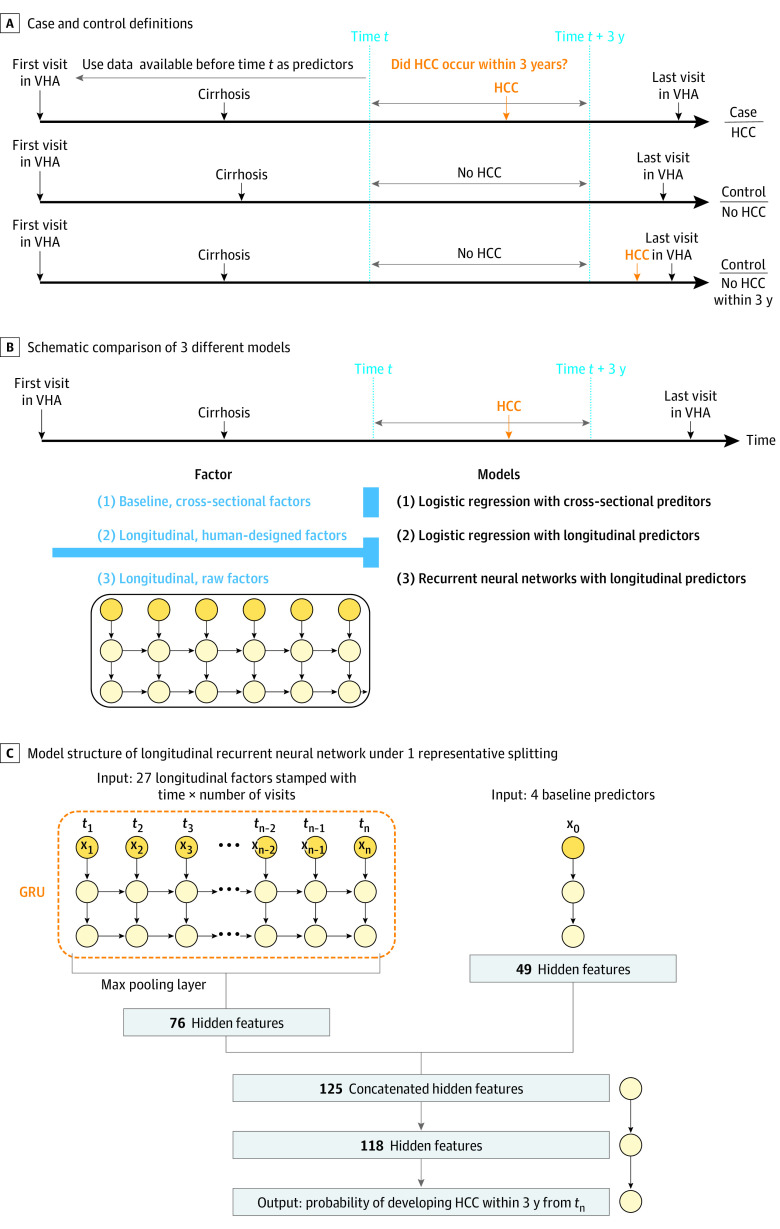
Schematics of Case and Control Definitions and Models Developed to Predict HCC Development A, Patients with hepatitis C virus infection who had a diagnosis of cirrhosis and at least 3 years of follow-up from the time of diagnosis of cirrhosis to their last follow-up visit in the Veterans Healthcare Administration (VHA) were identified. Patients who developed hepatocellular carcinoma (HCC) within 3 years of time *t *after the development of cirrhosis were designated cases, and those who did not were designated controls. All data available at or before time *t* were used as predictors of the development of cirrhosis within 3 years of time *t*. The first and third examples are for patients who developed HCC during follow-up; the second example is for a patient who did not develop HCC during follow-up. B, Schematic comparison of the 3 different models we developed to predict HCC development (ie, model 1, logistic regression using cross-sectional baseline data at time *t*; model 2, logistic regression using human-designed longitudinal data prior to time *t*; and model 3, recurrent neural networks using raw longitudinal data prior to time *t*). C, Model structure of longitudinal recurrent neural network under 1 representative splitting.

We wanted to simulate the clinically relevant scenario in which a physician would like to estimate the probability that a specific patient with cirrhosis will develop HCC within the following 3 years from the time of the clinic visit using all available information at the time of the clinic visit. To do this we analyzed cases and controls by sampling random clinic visits as follows (Figure 1A).

#### Cases

We identified 10 738 patients who developed HCC during follow-up and had at least 1 visit after the diagnosis of cirrhosis within 3 years before the diagnosis of HCC. We randomly sampled 1 visit (time *t*) for each patient. Thus, we obtained 10 738 case samples in which HCC was diagnosed within 3 years of the sampled visit (time *t*) (Figure 1A).

#### Controls

For patients who did not develop HCC during follow-up (n = 37 410), we randomly sampled 1 visit (time *t*) after the diagnosis of cirrhosis that was at least 3 years before the date of last follow-up for each patient to ensure that no HCC developed within 3 years (Figure 1A). In addition, we wanted to capture visits of patients who developed HCC more than 3 years after time *t* as controls. We identified 4835 patients who had more than 3 years of follow-up from the date of cirrhosis diagnosis to the date of HCC diagnosis and included 1 control visit that occurred more than 3 years before the diagnosis of HCC (Figure 1A). Thus, we obtained 42 245 control samples, in which HCC was not diagnosed within 3 years of the sampled visit (time *t*). This resulted in 52 983 total samples from 48 151 patients.

### Model Building and Feature Extraction

We developed and compared 3 different models predicting the probability of developing HCC within 3 years after time *t* (Figure 1B). First, we developed LR models using only the baseline, cross-sectional (cross-sectional LR) values of each of the predictors immediately prior to time *t*, which assumes the linearity of these variables and log odds. Sex, race, and HCV genotype were modeled as dummy categorical variables. Laboratory tests were modeled as continuous variables. We imputed missing values by the mean of nonmissing entries in the training data.

Second, we developed LR models as described earlier; however, we included the following 5 summary statistics for each of the longitudinal predictors (longitudinal LR), designed to capture longitudinal information available prior to time *t*: minimum, maximum, minimum of slope, maximum of slope, and total variation. The slope is defined as the ratio of difference of longitudinal predictor and the time gap between 2 consecutive visits. The total variation is defined as the mean of absolute value of slopes. We performed feature selection for the cross-sectional LR and longitudinal LR models using the lasso approach.^32^

Third, we developed an RNN model that can use information from both the baseline predictors and the raw longitudinal predictors from the first visit in the VA until the time of visit (Figure 1C). We imputed missing values by filling the missing entries at the first visit by the mean of nonmissing entries of training data and by replacing the remaining missing entries at any time by looking backward, ie, the latest available nonmissing values prior to this time.

In contrast to conventional LR, which requires specific feature extraction, RNNs can handle a varied number of visits and irregular time gaps between 2 consecutive visits. It can also automatically learn features that are useful for prediction. Specifically, we used gated recurrent units,^33^ an improved version of standard RNN that can better store long-term information. After combining temporal information from longitudinal predictors and time-invariant information from baseline predictors, we constructed a classifier using feedforward neural networks.^34^ We used the Rectified Linear Unit (ReLU)^35^ as the nonlinear activation function and used the sigmoid activation to return a risk probability between 0 and 1 in the output layer. To prevent overfitting, a dropout layer was added after a nonlinear activation.^36^ All parameters were optimized through minimizing the binary cross-entropy loss with the Adam stochastic algorithm.^37^

### Statistical Analysis

We randomly split the cohort into a training set (90%) and a testing set (10%). We fit 3 models using the training set and evaluated prediction performances using the same testing set. We repeated this procedure 10 times and reported the mean performance characteristics on the testing set over 10 random splits. We report 2-sided *P *values of the paired sample *t* test when comparing the performance characteristics between LR models and the RNN model. Statistical significance was set at *P* < .05.

Performance characteristics to evaluate the models’ discrimination were assessed based on the area under the receiver operating characteristic curve (AUROC) and the area under the precision-recall curve (AUPRC).^38^ We used the Brier score^39^ to compare overall accuracy; a Brier score of 0 signifies perfect accuracy. Furthermore, we provided the mean of predicted probability and the proportion of actual positive outcomes for 3 risk categories according to thirds of predicted risk for each model to calibrate the probabilities; a well-calibrated model should obtain similar results for each category.

For the LR models with lasso penalty, a hyperparameter tuning was done by first identifying the optimal penalty coefficient based on a 5-fold cross-validation, and then the model was fit with the selected coefficient using the training set. For the RNN model, we fixed some hyperparameters to reduce the computational cost (hidden layers set at 2; dropout rate, 0.2; batch size, 256). We searched for the optimal hidden sizes of model structures because they are more sensitive in prediction performance based on our evaluation. We performed LR models without lasso penalty using the Scikit-learn library^40^ in Python version 3.45.7 and LR models with lasso penalty by the glmnet R version 3.6.1 package^41^ (R Project for Statistical Computing). The numerical implementation of the RNN model was in PyTorch version 1.1.^42^

## Results

### Characteristics of the Population Used for Model Building

Of 52 983 samples, most came from men (51 948 [98.0%]), as expected in a VHA population, with representation from multiple racial and ethnic groups (Table 1). Compared with control samples from patients who did not develop HCC within 3 years of the sampled visit, patients who developed HCC within 3 years were older when they were diagnosed with cirrhosis (mean [SD] age, 56.9 [6.9] years vs 58.2 [6.6] years), were more likely to have genotype 3 HCV (3256 [7.7%] vs 1212 [11.3%]), were less likely to achieve SVR (5680 [13.4%] vs 1192 [11.1%]), had been diagnosed with cirrhosis for a longer time (mean [SD] duration of cirrhosis diagnosis, 1.97 [2.61] years vs 2.72 [3.30] years), had higher serum AST, ALT, and bilirubin levels (mean [SD] AST: 71.7 [47.2] U/L vs 87.1 [49.5] U/L [to convert to microkatals per liter, multiply by 0.0167]; mean [SD] ALT, 70.1 [71.0] U/L vs 74.9 [58.5] U/L [to convert to microkatals per liter, multiply by 0.0167]; mean [SD] bilirubin, 1.2 [1.5] mg/dL vs 1.5 [1.5] mg/dL [to convert to millimoles per liter, multiply by 17.104]), had higher FIB-4 and APRI scores (mean [SD] FIB-4 score: 5.1 [4.7] vs 6.8 [5.2]; mean [SD] APRI score: 1.8 [2.0] vs 2.5 [2.2]), and had a lower mean (SD) platelet count (141.1 [74.5] ×10^3^/μL vs 121.0 [68.3] ×10^3^/μL [to convert to ×10^9^ per liter, multiply by 1.0]) at the sampled visit (time *t*).

**Table 1.** Characteristics of Controls and Cases Used in Model Building at the Sampled Visit

Characteristic	Mean (SD)	
	Samples of patients who did not develop HCC within 3 y (n = 42 245)	Samples of patients who developed HCC within 3 y (n = 10 738)
Age at cirrhosis diagnosis, y	56.9 (6.9)	58.2 (6.6)
Men, No. (%)	41 315 (97.8)	10 633 (99.0)
Race/Ethnicity, No. (%)		
White, non-Hispanic	23 681 (56.1)	5996 (55.8)
Black, non-Hispanic	10 805 (25.6)	2626 (24.5)
Hispanic, Asian, Pacific Island, AIAN, or other	4956 (11.7)	1323 (12.35)
Declined to answer or missing	2803 (6.6)	793 (7.4)
Genotype, No. (%)		
1	30 702 (72.7)	7497 (69.8)
2	3289 (7.8)	637 (5.9)
3	3256 (7.7)	1212 (11.3)
≥4	356 (0.8)	93 (0.9)
Missing	4642 (11.0)	1299 (12.1)
Achieved SVR at time *t*, No. (%)	5680 (13.4)	1192 (11.1)
Duration of cirrhosis at time *t*, y	1.97 (2.61)	2.72 (3.30)
BMI at time *t*	28.7 (5.6)	28.1 (5.4)
Laboratory test results at time *t*		
AST, U/L	71.7 (47.2)	87.1 (49.5)
ALT, U/L	70.1 (71.0)	74.9 (58.5)
Platelet count, ×10^3^/μL	141.1 (74.5)	121.0 (68.3)
Bilirubin, mg/dL	1.2 (1.5)	1.5 (1.5)
INR	1.2 (0.3)	1.2 (0.3)
Creatinine, mg/dL	1.1 (1.0)	1.0 (0.7)
FIB-4 score	5.1 (4.7)	6.8 (5.2)
APRI score	1.8 (2.0)	2.5 (2.2)

Abbreviations: AIAN, American Indian/Alaskan Native; ALT, alanine aminotransferase; AST, aspartate transaminase; APRI, AST to platelet ratio index; BMI, body mass index (calculated as weight in kilograms divided by height in meters squared); FIB-4, fibrosis-4; INR, international normalized ratio; SVR, sustained virologic response.

SI conversion factors: To convert ALT and AST to microkatals per liter, multiply by 0.0167; bilirubin to millimoles per liter, multiply by 17.104; creatinine to millimoles per liter, multiply by 88.4; and platelet count to ×10^9^ per liter, multiply by 1.0.

### Model Performance Among All Samples

**Figure 2.**
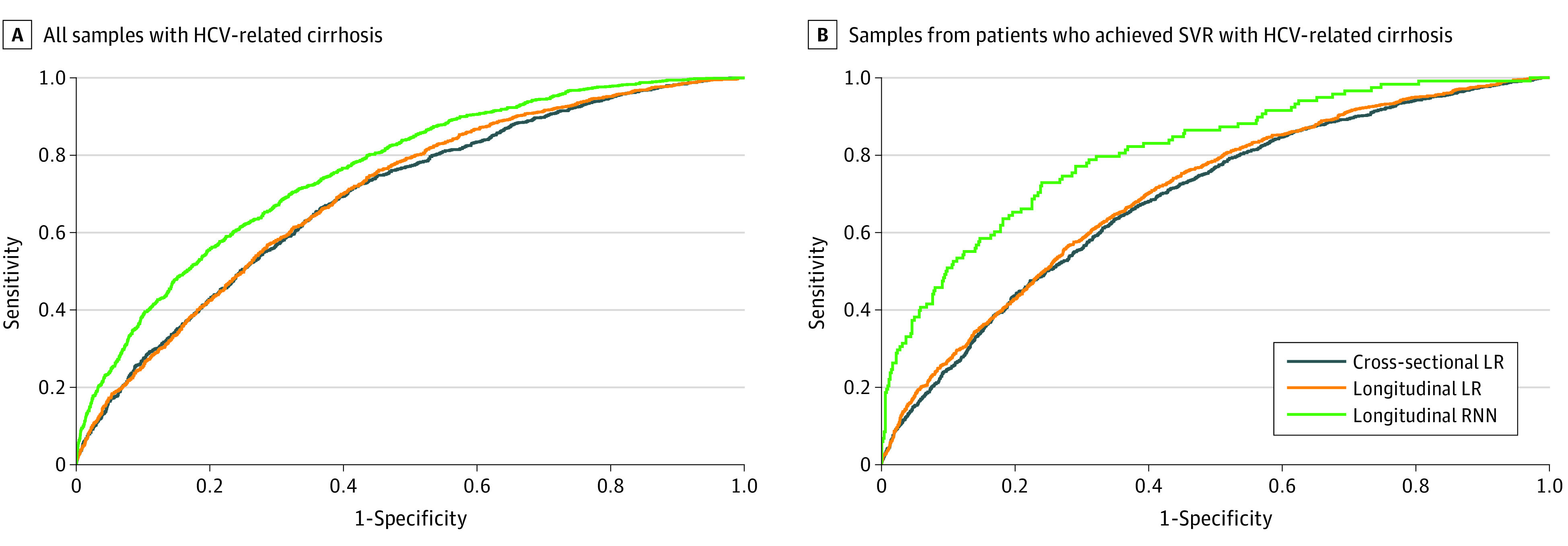
Receiver Operating Characteristic Curves for 3 Prediction Models We developed 3 different models predicting the development of hepatocellular carcinoma within 3 years under 1 representative splitting (results based on the testing set) in all samples from patients with hepatitis C virus (HCV)–related cirrhosis and samples from patients who achieved sustained virologic response (SVR) with HCV-related cirrhosis.

The RNN model resulted in significantly higher mean (SD) AUROC (0.759 [0.009]), a measure of discrimination, than the longitudinal LR (0.689 [0.009]) or cross-sectional LR (0.682 [0.007]) models without feature selection (*P* < .001 for both comparisons) (Table 2 and Figure 2A). The absolute value of the AUROC achieved by the RNN model is considered good. The RNN model achieved significantly higher mean (SD) AUPRC (0.479 [0.018]) than the longitudinal LR (0.361 [0.009]) or cross-sectional LR (0.345 [0.011]) models without feature selection (*P* <.001 for both comparisons). Also, the RNN model resulted in a significantly lower mean (SD) Brier score (0.136 [0.003]), a measure of overall accuracy, than the longitudinal LR (0.149 [0.003) or cross-sectional LR (0.150 [0.003]) models without feature selection (*P* <.001 for both comparisons). In comparison with the longitudinal LR model that used specific summary statistics of longitudinal predictors, the RNN model obtained significant improvement by automatically extracting useful features from raw longitudinal predictors.

**Table 2.** Comparison of the Performance Characteristics of 3 Different Models Predicting the Development of Hepatocellular Carcinoma Within 3 Years in Patients With Hepatitis C Virus–Related Cirrhosis

Performance characteristic	Mean (SD)			*P* value compared with RNN model
	Cross-sectional LR model	Longitudinal LR model	RNN model	
**All samples**				
AUROC	0.682 (0.007)	0.689 (0.009)	0.759 (0.009)	<.001
Brier score	0.150 (0.003)	0.149 (0.003)	0.136 (0.003)	<.001
AUPRC	0.345 (0.011)	0.361 (0.009)	0.479 (0.018)	<.001
Proportion of samples who test positive at 90% sensitivity	0.746 (0.008)	0.736 (0.013)	0.663 (0.012)	<.001
Specificity at 90% sensitivity	0.293 (0.010)	0.305 (0.016)	0.397 (0.014)	<.001
Positive predictive value at 90% sensitivity	0.243 (0.003)	0.246 (0.003)	0.273 (0.006)	<.001
Negative predictive value at 90% sensitivity	0.920 (0.006)	0.923 (0.007)	0.940 (0.003)	<.001
Proportion of samples who test positive at 80% sensitivity	0.601 (0.012)	0.591 (0.017)	0.514 (0.015)	<.001
Specificity at 80% sensitivity	0.449 (0.015)	0.462 (0.021)	0.558 (0.018)	<.001
Positive predictive value at 80% sensitivity	0.268 (0.007)	0.27 (30.009)	0.314 (0.009)	<.001
Negative predictive value at 80% sensitivity	0.898 (0.004)	0.901 (0.005)	0.916 (0.004)	<.001
**Samples from patients who achieved SVR**				
AUROC	0.672 (0.030)	0.705 (0.024)	0.806 (0.025)	<.001
Brier score	0.139 (0.006)	0.136 (0.006)	0.117 (0.007)	<.001
AUPRC	0.333 (0.060)	0.361 (0.050)	0.519 (0.064)	<.001
Proportion of samples who test positive at 90% sensitivity	0.793 (0.041)	0.702 (0.028)	0.571 (0.052)	<.001
Specificity at 90% sensitivity	0.230 (0.050)	0.340 (0.035)	0.499 (0.064)	<.001
Positive predictive value at 90% sensitivity	0.205 (0.022)	0.230 (0.020)	0.285 (0.033)	<.001
Negative predictive value at 90% sensitivity	0.904 (0.013)	0.933 (0.006)	0.954 (0.005)	<.001
Proportion of samples who test positive at 80% sensitivity	0.628 (0.040)	0.559 (0.035)	0.429 (0.039)	<.001
Specificity at 80% sensitivity	0.409 (0.051)	0.492 (0.043)	0.651 (0.047)	<.001
Positive predictive value at 80% sensitivity	0.230 (0.026)	0.257 (0.023)	0.337 (0.036)	<.001
Negative predictive value at 80% sensitivity	0.898 (0.009)	0.914 (0.009)	0.934 (0.007)	<.001

Abbreviations: AUPRC, area under the precision-recall curve; AUROC, area under receiver operating characteristic curve; LR, logistic regression; RNN, recurrent neural network; SVR, sustain virologic response.

For the 2 LR models with feature selection, the lasso method only eliminated a small mean number of features and resulted in very similar AUROC, AUPRC, and Brier scores. These scores were inferior to those of the RNN model (eTable 2 in the Supplement).

### Prioritizing Patients for HCC Screening Outreach Interventions

We envision that risk stratification models could be used to prioritize the patients with the highest risk for screening outreach interventions. Using the RNN model, we determined that 90% of all HCC diagnoses in the following 3 years occurred in samples with the mean (SD) highest 66% (1.2%) of risk scores, whereas 80% of HCCs occurred in samples with the mean (SD) highest 51% (1.5%) of risk scores. Thus, using the RNN model, we could potentially target the top 51% of samples with the highest HCC risk scores, in which 80% of all HCCs occurred, or the top 66% of samples with the highest HCC risk scores, in which 90% of all HCCs occurred. In contrast, the proportions that would need to be screened to include 80% or 90% of patients who would be diagnosed with HCC were much greater using the longitudinal LR and cross-sectional LR models (Table 2).

### Model Calibration

All 3 models had excellent agreement between observed and predicted 3-year risks when the population was divided into 3 risk categories according to thirds of predicted risk for each model (Table 3), implying that all models were well calibrated. However, the RNN model achieved much greater separation between the first and third tertiles (predicted 3-year HCC risk 2% vs 25%) than the longitudinal LR (4% vs 19%) or cross-sectional LR (5% vs 18%) models.

**Table 3.** Comparison of Predicted and Observed 3-Year HCC Risk in the Study Population Divided Into Tertiles According to Each Model Under 1 Representative Splitting^a^

Tertile	Cross-sectional LR model			Longitudinal LR model			RNN model		
	%		No. (%)	%		No. (%)	%		No. (%)
	Observed 3-y HCC risk	Predicted 3-y HCC risk		Observed 3-y HCC risk	Predicted 3-y HCC risk		Observed 3-y HCC risk	Predicted 3-y HCC risk	
First, low risk	4	5	17661 (33.3)	4	4	17661 (33.3)	2	2	17661 (33.3)
Second, medium risk	10	9	17661 (33.3)	9	9	17661 (33.3)	7	8	17661 (33.3)
Third, high risk	18	18	17661 (33.3)	18	19	17661 (33.3)	24	25	17661 (33.3)

Abbreviations: HCC, hepatocellular carcinoma; LR, logistic regression; RNN, recurrent neural network.

^a^ The observed 3-year HCC risk is the proportion of those who developed HCC within 3 years among samples in each group. The predicted 3-year HCC risk is the mean of probabilities that are returned by models for samples in each group.

### Model Performance Among Samples From Patients Who Achieved SVR and Men

Because most patients with HCV infection now undergo treatment with direct-acting antivirals and achieve SVR that reduces the risk of HCC, we evaluated our models’ performance characteristics among a subset of samples from patients who achieved SVR during follow-up. Mean (SD) AUROC (0.806 [0.025]), AUPRC (0.519 [0.064]), and Brier score (0.117 [0.007]) of the RNN model (Table 2 and Figure 2B) were all superior in the subset who achieved SVR than in the entire population; these scores continued to be superior to the LR models.

We further evaluated 3 models among samples from male patients, given that 98% of patients with HCV infection patients in the VA system were men. The performance characteristics of the RNN model on samples from male patients were very similar to those on the entire population and continued to be superior to the LR models (eTable 3 in the Supplement).

## Discussion

The past decade has seen an explosion in the amount of medical information stored in electronic health records (EHRs). Such EHR data are potentially ideal for deep learning algorithms, but surprisingly few applications of deep learning have been developed that use EHR data to assist with diagnosis or prognosis.^43^ We demonstrated an application for RNN models that outperformed conventional LR models in the prediction of HCC risk in patients with HCV-related cirrhosis, including those who achieve SVR following antiviral therapy.

Changes in many predictor variables over time can provide crucial prognostic information, but such changes are difficult or impossible to model using conventional regression modeling algorithms. RNNs are powerful methods for processing sequential data and have shown superior performance in many applications, such as machine translation.^44^ The specific structure of RNNs can handle temporal data with varying length and capture long-term dependencies, which enables automatic feature learning needed for prediction from raw temporal data. This is especially important because it does not depend on human-engineered feature extraction and discovers novel patterns using all information within the analysis.

Our RNN models exhibited an AUROC of 0.759 among all samples and 0.806 among samples from patients with SVR, which is considered very good and compares favorably with other HCC risk prediction models.^6,45,46^ Equally importantly, our models had great calibration and excellent agreement between observed and predicted HCC risk (Table 3).

We envision 2 areas of clinical implementation of HCC risk prediction models such as the RNN models we developed, aimed at improving HCC surveillance strategies. First, our models can be used to improve screening outreach efforts. Currently, less than 50% of patients with cirrhosis get regular HCC surveillance across most health care systems.^47,48^ RNN models could be used to identify the patients with the highest risk, who could then be targeted for interventions to improve their uptake of HCC surveillance. For example, we demonstrated that by targeting the samples with the top 51% of HCC risk scores calculated by our RNN models, we would be including 80% of patients who would develop HCC in the next 3 years, while targeting the top 66% would include 90% of patients who would develop HCC. This is a much more effective strategy than our current first-come, first-served approach to outreach for HCC screening. Identifying and offering screening only to the patients with the highest risk could also be a plausible strategy in health care systems around the world that do not have the capacity to screen all at-risk patients. Assuming that a given maximum number of screening studies can be performed in such health care systems, these studies will lead to early diagnosis of HCC in a higher proportion of patients if they are targeted to patients with the highest HCC risk identified by our RNN models than if they are randomly distributed among all patients with cirrhosis.

Second, we envision that our RNN models could also be used in the future to identify high-risk patients for new surveillance strategies that are more effective than the current strategy of ultrasonography and α-fetoprotein, but are also more expensive (ie, risk-based screening). Many new surveillance strategies are being investigated in phase 2 and 3 studies and some are already available in clinical practice. For example, abbreviated magnetic resonance imaging protocols have been developed specifically for the purposes of HCC screening, which have much greater sensitivity and specificity than ultrasonography.^49,50,51^ However, these examinations are substantially more expensive than ultrasonograph examinations and would have to be limited to high-risk patients. Also, multiple novel biomarker panels are being developed that could also be more cost-effective if they were combined with ultrasonography in high-risk patients.

Given that most patients with HCV infection are now expected to undergo treatment and achieve SVR and given that SVR reduces the risk of HCC, it is imperative that HCC risk prediction models incorporate SVR and predict well among patients who achieve SVR. Indeed, the performance of our RNN models was even better among samples from patients who achieved SVR than among the entire population.

### Limitations

This study has limitations related to lack of external validation and the computational cost of running the analyses. To reduce computational cost, we only performed optimal search for some of the hyperparameters. Even so, the RNN model outperformed conventional LR models. Health care systems are now investing in the infrastructure to construct some of these complex models. For example, the VHA has collaborated with Google’s DeepMind to develop an RNN model for predicting acute kidney injury using national VHA data.^43^ All deep learning neural network models, including ours, have limited interpretability due to their black-box nature, which may limit acceptability by clinicians. However, recent innovations allow for interpretable deep learning models by determining the proportion of the prediction attributed to each feature.^52,53^

## Conclusions

In this study, we demonstrated that RNN models that use raw longitudinal EHR data are superior to conventional LR models in estimating the risk of HCC in patients with HCV-related cirrhosis. RNN models such as ours could have multiple applications in clinical practice, provided they can be incorporated within EHR software systems.

## Notes

Supplement. **eTable 1. ***ICD-10* and *ICD-9* Codes for Cirrhosis, Decompensated Cirrhosis, and HCC **eTable 2. **Feature Selection Frequency Over 10 Splits for the Longitudinal LR With Lasso Penalty **eTable 3. **Comparison of the Performance Characteristics of 3 Different Models Predicting the Development of HCC Within 3 years in Male Patients with HCV-Related Cirrhosis
