# Supplementary material for: Assessment of a Deep Learning Model to Predict Hepatocellular Carcinoma in Patients With Hepatitis C Cirrhosis
Source: JAMA Netw Open. 2020 Sep 1;3(9):e2015626. doi: 10.1001/jamanetworkopen.2020.15626 (PMC7489819; doi:10.1001/jamanetworkopen.2020.15626)
Supplement: Supplement. — eTable 1. ICD-10 and ICD-9 Codes for Cirrhosis, Decompensated Cirrhosis, and HCC eTable 2. Feature Selection Frequency Over 10 Splits for the Longitudinal LR With Lasso Penalty eTable 3. Comparison of the Performance Characteristics of 3 Different Models Predicting the Development of HCC Within 3 years in Male Patients with HCV-Related Cirrhosis [file jamanetwopen-e2015626-s001.pdf]

Supplementary Online Content

Ioannou GN, Tang W, Beste LA, et al. Assessment of a deep learning model to predict hepatocellular carcinoma in patients with hepatitis C cirrhosis. *JAMA Netw Open*. 2020;3(9):e2015626. doi:10.1001/jamanetworkopen.2020.15626

eTable 1. *ICD-10* and *ICD-9* Codes for Cirrhosis, Decompensated Cirrhosis, and HCC

eTable 2. Feature Selection Frequency Over 10 Splits for the Longitudinal LR With Lasso Penalty

eTable 3. Comparison of the Performance Characteristics of 3 Different Models Predicting the Development of HCC Within 3 years in Male Patients with HCV-Related Cirrhosis

This supplementary material has been provided by the authors to give readers additional information about their work.

© 2020 Ioannou GN et al. *JAMA Network Open.*

eTable 1. *ICD-10* and *ICD-9* Codes for Cirrhosis, Decompensated Cirrhosis, and HCC

**Diagnosis**	**ICD9**		**ICD10**
**HEPATOCELLULAR CARCINOMA**	155.0	**HEPATOCELLULAR CARCINOMA**	C22.0
**CIRRHOSIS**			
Alcoholic Cirrhosis Of Liver	571.2	Alcoholic cirrhosis without ascites	K70.30
Cirrhosis Of Liver Without Mention Of Alcohol	571.5	Other cirrhosis of liver	K74.69
		Unspecified cirrhosis of the liver	K74.60
		Pigmentary cirrhosis of the liver	E83.110
		Cirrhosis (of liver) with toxic liver disease	K71.7
**DECOMPENSATED CIRRHOSIS (INCLUDES ANY OF THE FOLLOWING)**			
**VARICES NO BLEEDING**			
Esophageal Varices Without Mention Of Bleeding	456.1	Esophageal varices, no bleeding	I85.00
Esophageal Varices In Diseases Classified Elsewhere, Without Mention Of Bleeding	456.21	Gastric Varices, no bleeding	I86.40
		Secondary esophageal varices, no bleeding	I85.10
**VARICES WITH BLEEDING**			
Esophageal Varices With Bleeding	456.0	Esophageal varices, with bleeding	I85.01
Esophageal Varices In Diseases Classified Elsewhere, With Bleeding	456.20	Gastric Varices, with bleeding	I86.41
		Secondary esophageal varices, with bleeding	I85.11
**ASCITES**			
Ascites	789.5	Alcoholic cirrhosis with ascites	K70.31
Other ascites	789.60	Ascites in alcoholic hepatitis	K70.11
Non-malignant ascites	789.59	Ascites in toxic liver disease with chronic active hepatitis	K71.51
		Other ascites	R18.8
**SPONTANEOUS BACTERIAL PERITONITIS**	567.23		K65.2
**ENCEPHALOPATHY***			
Encephalopathy	572.2	Hepatic failure, unspecified with coma	K72.91
Chronic hepatitis C with hepatic coma		Encephalopathy, unspecified	G93.40
		Chronic failure with coma	K72.11
**HEPATORENAL SYNDROME**	572.5		K76.7
**HEPATOPULMONARY SYNDROME**	573.5		K76.81

© 2020 Ioannou GN et al. *JAMA Network Open.*

eTable 2. Feature Selection Frequency Over 10 Splits for the Longitudinal LR With Lasso Penalty[1]

Feature	Selection frequency	Feature	Selection frequency	Feature	Selection frequency
genotype_main_>=4	10	alkalinephosphatase_max	10	hemoglobin_maxdiff	9
alt_maxdiff	10	alkalinephosphatase_min	10	totprotein_max	9
potassium_tv	10	alkalinephosphatase_mindiff	10	ASTALT_min	9
glucose_max	10	alkalinephosphatase_maxdiff	10	albumin_tv	9
glucose_min	10	APRI_min	10	alkalinephosphataseratio_min	9
glucose_tv	10	APRI_tv	10	wbc_tv	9
plt_max	10	hemoglobin_tv	10	alphafetoprotein_mindiff	9
plt_min	10	sodium_max	10	Fib4_min	9
plt_mindiff	10	BMI_tv	10	alphafetoproteinratio_min	9
alt_max	10	inr_mindiff	10	bilirubin_max	9
alt_mindiff	10	Race_HISPANIC	10	Race_WHITE	9
alphafetoprotein_max	10	bilirubin_mindiff	10	glucose_mindiff	8
potassium_mindiff	10	Race_BLACK OR AFRICAN AMERICAN	10	glucose_maxdiff	8
alphafetoprotein_maxdiff	10	inr_max	10	Fib4_mindiff	8
genotype_main_3	10	AgeAtCirrhosis	10	APRI_max	8
bloodureanitro_max	10	inr_min	10	totprotein_maxdiff	8
bloodureanitro_min	10	SVR	10	Fib4_maxdiff	8
bloodureanitro_mindiff	10	BMI_maxdiff	10	inr_maxdiff	8
bloodureanitro_maxdiff	10	albumin_maxdiff	10	alkalinephosphataseratio_max	8
hemoglobin_max	10	albumin_min	10	alphafetoproteinratio_maxdiff	8
hemoglobin_min	10	albumin_max	10	alkalinephosphatase_tv	7
hemoglobin_mindiff	10	altratio_min	10	APRI_mindiff	7
potassium_maxdiff	10	wbc_min	10	Fib4_tv	7
potassium_min	10	wbc_maxdiff	10	wbc_mindiff	7
ASTALT_tv	10	Race_OTHER	10	alkalinephosphataseratio_maxdiff	7
chloride_mindiff	10	bilirubin_tv	10	alt_min	7
sodium_min	10	genotype_main_2	10	ast_mindiff	7
sodium_mindiff	10	Gender_M	10	plt_tv	6
sodium_maxdiff	10	BMI_min	10	alphafetoprotein_min	6
sodium_tv	10	ast_min	10	bilirubin_min	6
Fib4_max	10	BMI_max	10	astratio_maxdiff	6
creatinine_max	10	ast_tv	10	BMI_mindiff	6
creatinine_min	10	genotype_main_1	10	creatinine_tv	6
chloride_max	10	ast_maxdiff	10	inr_tv	6
chloride_min	10	ast_max	10	ASTALT_max	6
chloride_maxdiff	10	Gender_F	10	altratio_max	6
potassium_max	10	totprotein_mindiff	10		
totprotein_min	10	totprotein_tv	10		

[1]For two logistic regression models with feature selection, 36 out of the original 37 features were selected for the cross-sectional-LR and 112 out of the original 137 features were selected for the longitudinal-LR. The prediction performance

© 2020 Ioannou GN et al. *JAMA Network Open.*

of two logistic regression models with lasso penalty is very close to that of models without lasso penalty - the average AUROC, AUPRC and Brier score of the longitudinal-LR were 0.689, 0.353 and 0.149; those of the cross-sectional-LR were 0.682, 0.344 and 0.150. The RNN model achieved better prediction performance than two logistic regression models either with feature selection or without feature selection.

© 2020 Ioannou GN et al. *JAMA Network Open.*

eTable 3. Comparison of the Performance Characteristics of 3 Different Models Predicting the Development of HCC Within 3 years in Male Patients with HCV-Related Cirrhosis

MALE PATIENTS	**Cross-sectional LR Model**	**Longitudinal LR Model**	**RNN Model**	**P-value compared to RNN model**
AUROC	0.681±0.008	0.688±0.010	0.757±0.009	<0.0001
Brier score	0.151±0.003	0.150±0.003	0.137±0.003	<0.0001
AUPRC	0.346±0.011	0.362±0.009	0.481±0.019	<0.0001
Proportion of patients who test positive at 90% sensitivity	0.749±0.007	0.739±0.013	0.667±0.012	<0.0001
Specificity at 90% sensitivity	0.289±0.009	0.302±0.016	0.392±0.014	<0.0001
Positive predictive value at 90% sensitivity	0.244±0.006	0.248±0.007	0.274±0.005	<0.0001
Negative predictive value at 90% sensitivity	0.918±0.003	0.921±0.004	0.938±0.003	<0.0001
Proportion of patients who test positive at 80% sensitivity	0.602±0.012	0.594±0.017	0.515±0.016	<0.0001
Specificity at 80% sensitivity	0.447±0.015	0.458±0.021	0.558±0.019	<0.0001
Positive predictive value at 80% sensitivity	0.270±0.007	0.274±0.009	0.3165±0.010	<0.0001
Negative predictive value at 80% sensitivity	0.897±0.005	0.899±0.005	0.916±0.004	<0.0001

© 2020 Ioannou GN et al. *JAMA Network Open.*
